# Impact of Antibiotic Therapy on the Upper Respiratory Tract and Gut Mycobiome in Patients with Cystic Fibrosis

**DOI:** 10.3390/jof11090631

**Published:** 2025-08-28

**Authors:** Cristina Zubiria-Barrera, Malena Bos, Robert Neubert, Jenny Fiebig, Michael Lorenz, Michael Hartmann, Jochen G. Mainz, Hortense Slevogt, Tilman E. Klassert

**Affiliations:** 1 Department of Respiratory Medicine and Infectious Diseases, Medical School Hannover (MHH), German Center for Lung Research (DZL), Research Network “BREATH”, 30625 Hannover, Germany; malena.bos@helmholtz-hzi.de (M.B.); robert.neubert@helmholtz-hzi.de (R.N.); fiebig.jenny@mh-hannover.de (J.F.); slevogt.hortense@mh-hannover.de (H.S.); tilman.klassert@helmholtz-hzi.de (T.E.K.); 2 Dynamics of Respiratory Infections, Helmholtz Centre for Infection Research, 38124 Braunschweig, Germany; 3 Cluster of Excellence RESIST (EXC 2155), Hannover Medical School, Carl-Neuberg-Straße 1, 30625 Hannover, Germany; 4 Department of Pediatric Pulmonology/Allergology, Section Cystic Fibrosis Centre for Children and Adults, University Hospital Jena, 07747 Jena, Germany; michael.lorenz@med.uni-jena.de; 5 Hospital Pharmacy, University Hospital Jena, 07747 Jena, Germany; michael.hartmann@med.uni-jena.de; 6 Cystic Fibrosis Center, Klinikum Westbrandenburg, Brandenburg Medical School (MHB) University, 16816 Neuruppin, Germany; j.mainz@uk-brandenburg.de

**Keywords:** cystic fibrosis, healthy, mycobiome, antibiotics, *Candida* spp.

## Abstract

Patients with cystic fibrosis (CF) are frequently exposed to antibiotic treatments, which can alter the fungal communities (mycobiome) across their mucosal sites. This pilot study investigated the impact of antibiotic exposure on the mycobiome by analyzing fungal community dynamics in the upper respiratory- (nasal lavage) and gastrointestinal- (stool samples) tracts of 12 patients with CF following (a) long-term antibiotic treatment over a three-year period and (b) short-term antibiotic therapy during acute pulmonary exacerbations. Mycobiome profiles of the samples obtained from 38 healthy individuals were also analyzed and used for comparison purposes. The *ITS1* region of the fungal *rRNA* gene cluster was sequenced to characterize and quantify the fungal community composition in both cohorts. Compared to healthy controls, samples from the patients with CF who had undergone long-term antibiotic treatment revealed a significantly increased fungal biomass in both sino-nasal and stool samples. Moreover, diversity metrics revealed significant differences in nasal lavage samples, whereas the stool samples showed no significant variation. *Candida* spp. was significantly enriched in both nasal and stool samples from CF patients. Further analyses demonstrated a strong positive correlation between the relative abundance of *Candida* spp. and the cumulative antibiotic intake over the three-year period in sino-nasal samples, but not in stool samples. Acute antibiotic treatment during a pulmonary exacerbation episode also led to a marked increase in the abundance of *Candida* spp. in sino-nasal samples. These findings highlight the increased sensitivity of the sino-nasal mycobiome to both chronic and acute antibiotic exposure in CF patients, as characterized by a site-specific fungal overgrowth, particularly of *Candida* spp.

## 1. Introduction

Cystic fibrosis (CF) is a hereditary genetic disorder caused by autosomal recessive mutations in the CF transmembrane conductance regulator (CFTR) gene [1]. These mutations result in the functional impairment of exocrine tissues, leading to clinical multiorgan manifestations that may critically affect the respiratory and digestive tracts. This mostly includes exocrine and, later, endocrine pancreatic insufficiency, hepatobiliary disorders, and compromised mucociliary clearance within the upper and lower respiratory tract [2]. This impaired mucus clearance promotes early and persistent airway colonization by microbial communities [3,4,5], potentially leading to an inflammatory response that causes tissue damage and a gradual decline in the pulmonary function, with lung destruction being the primary reason for premature death in CF patients [6]. Opportunistic pathogens such as *Pseudomonas aeruginosa*, *Staphylococcus aureus*, and *Haemophilus influenzae* are common triggers of severe lung infections, such as pulmonary exacerbations. Moreover, repeated occurrences of these infections have been associated with rapid deterioration of lung function, which increases the mortality risk in CF patients [7,8]. Effective management of pulmonary infections involves targeted antibiotic therapy. Together with other therapeutic approaches, such as the recently approved CFTR-modulating therapies [9], antibiotics are crucial in controlling the progression of bacterial infections, minimizing exacerbation symptoms, preserving lung function, and improving the overall quality of life of these patients [10,11]. Nevertheless, growing evidence suggests that these therapies sometimes fail to fully eradicate the pathogens involved, which causes many patients to undergo frequent and, in some cases, lifelong antibiotic treatments [12]. In addition to recurrent bacterial infections, CF patients commonly experience fungal colonization. This predisposition likely results from several factors, including ineffective enhanced defense mechanisms, frequent antibiotic use, and repeated exposure to pathogenic microbes [6,13].

Much less is known about the fungal communities present in the lungs of healthy individuals. Based on studies on the bacterial microbiome of the respiratory tract, the healthy lung is believed to host a dynamic, transient, and diverse microbial environment. A balance is maintained between the spread of microbes from the upper respiratory tract into the lungs and the efficiency of the pulmonary microbial clearance mechanisms [14,15]. Some studies have reported that fungi such as *Aspergillus*, *Penicillium*, and *Cladosporium* species are commonly identified in the lungs of healthy individuals [16], likely originating from the continuous inhalation of environmental spores [17]. Non-filamentous fungi may also reach the lungs, through microaspiration, from the upper respiratory tract, where colonization is normally controlled by the host’s immunological and mechanical defenses [6]. When these defenses are impaired, microbial imbalance and persistent colonization of the respiratory tract may occur [13].

Studies investigating the composition of the lung mycobiome in respiratory diseases such as COPD, bronchiectasis, and asthma have frequently identified *Candida* spp. as one of the most dominant fungal genera in respiratory samples [18,19]. Despite its prevalence, the clinical significance of this opportunistic pathogen remains poorly understood and is often overshadowed by other fungi such as *Aspergillus fumigatus*, a well-known trigger of allergic bronchopulmonary aspergillosis [20]. Studies investigating the role of *Candida* spp. in patients with asthma have shown that this fungus can influence type 2 immune responses, potentially exacerbating allergic inflammation [21]. In patients with CF, respiratory samples often reveal a dominant fungal community, particularly of the *Candida* genus, alongside a diverse array of filamentous fungi such as *Aspergillus*, *Scedosporium*, and *Exophiala* species [22,23,24,25]. For instance, Muthig et al. reported persistent colonization of *Candida albicans* in the lower respiratory tract of CF patients over a 2-year study [26]. While certain fungi, such as *A. fumigatus*, are clearly associated with diseases like allergic bronchopulmonary aspergillosis and invasive aspergillosis, particularly in CF [24,27], the role of *Candida* spp. remains less defined. Some studies link *C. albicans* colonization in CF to reduced lung function and increased pulmonary exacerbations [28]. This suggests that, although *Candida* may not cause direct infection, its presence could contribute to disease severity by promoting inflammation or forming biofilms, especially with bacterial pathogens like *Pseudomonas aeruginosa* [29]. Overall, while the pathogenic role of *Candida* spp. in respiratory diseases is still unclear, it is consistently found as a prevalent member of the airway mycobiome.

Several factors might influence the chronic colonization of these microorganisms in the respiratory tract of CF patients. Notably, long-term use of inhaled antibiotics, i.e., tobramycin or colomycin, which are standard therapies in CF patients who are chronically colonized with *P. aeruginosa*, has been strongly associated with chronic colonization of *Candida* spp. in a one-year study [30], while intravenous antibiotics have been linked to a reduced presence of *Aspergillus* species in the lung of CF patients [31]. These findings suggest that bacterial eradication through antibiotic treatment may influence shifts in fungal communities; however, it remains unclear to what extent these changes correlate with short-term or long-term antibiotic exposure.

In this study, we aimed to investigate the effects of both long-term and short-term antibiotic treatment on the mycobiome profiles of the upper respiratory and gastrointestinal tracts in patients with CF, compared to healthy individuals. While previous research has primarily focused on the lung or sputum microbiomes, the site-specific impact of antibiotic therapy on fungal communities across different mucosal sites remains underexplored. By analyzing nasal lavage and stool samples, two clinically relevant, non-invasive sample types, we assessed fungal community dynamics during both stable periods and pulmonary exacerbations. To quantify and standardize the cumulative antibiotic exposure, we applied a custom-developed mathematical formula to calculate an equivalence-based score, which allowed us to measure the total antibiotic burden over time. This approach enabled the evaluation of the long-term impact of antibiotic use on mycobiome dynamics in both the upper respiratory and gastrointestinal tracts. Additionally, we examined short-term shifts in fungal communities by comparing samples collected at the onset of pulmonary exacerbations and after antibiotic therapy. Notably, all samples analyzed in this study were collected between 2018 and 2021, prior to the widespread introduction of the highly effective CFTR modulator triple therapy (elexacaftor/tezacaftor/ivacaftor) in Germany in mid-2021.

## 2. Materials and Methods

### 2.1. Study Population and Sample Collection

This study included 12 CF patients recruited from the Cystic Fibrosis Unit at the University Hospital Jena (Germany) between 2018–2021 (Table S1). Nasal lavage and stool samples were collected from each patient at three different time points: before and after an acute phase of pulmonary exacerbation and concomitant antibiotic treatment, and at a stable phase (three months after the last antibiotic treatment). Patients’ clinical data were recorded, including age, gender, body mass index (BMI), *CFTR* gene mutation class, spirometry values (FEV1%), treatments with CFTR-modulator therapies, use of topical as well as systemic corticosteroids, and antibiotic therapies administered over a three-year period prior to sample collection. For comparative analyses, nasal lavage and stool samples were collected from a cohort of 38 healthy volunteers who had not undergone antibiotic treatment in the six months prior to sample collection. From this cohort, 18 nasal lavage and 38 stool samples were obtained (Table S2).

Sino-nasal samples were collected using nasal lavage, as previously described [32,33]. Briefly, 10 mL of sterile isotonic saline was gently instilled into each nostril using a 10 mL syringe while the subject reclined their head with their soft palate closed. The isotonic solution was retained in the nasal cavities for approximately 10 s without swallowing, after which it was expelled by tilting the head forward, lightly exhaling, and allowing the lavage fluid to drain into a sterile plastic beaker. Stool samples were collected in tubes containing 9 mL of DNA/RNA Shield solution (Zymo Research, Irvine, CA, USA), which facilitates microbial lysis and stabilizes DNA. All samples were aliquoted and stored at −80 °C until further processing.

### 2.2. DNA Extraction and ITS1 Region Copy Number Quantification

Microbial DNA from nasal lavage samples was extracted using the ZymoBIOMICS DNA Miniprep Kit (Zymo Research, Irvine, CA, USA). DNA extraction included two mechanical lysis steps performed on a SpeedMill Plus (Analytik Jena, Jena, Germany) at 50 Hz for 2 × 3 min cycles. The first lysis step used 1.4 mm zirconium-silicate bead lysing tubes (MP Biomedicals, Irvine, CA, USA), and was followed by a second step using a mixture of 0.1 mm and 0.5 mm BashingBeads (Zymo Research, Irvine, CA, USA) provided in the extraction kit. Subsequent steps were carried out according to the manufacturer’s protocol.

DNA from stool samples was extracted using the innuPREP Stool DNA Kit (Analytik Jena, Jena, Germany), following the manufacturer’s protocol. An initial mechanical lysis step was added in which lysing tubes containing 1.4 mm zirconium-silicate beads were used (MP Biomedicals, Irvine, CA, USA).

Fungal biomass was quantified by qPCR, using 4 µL DNA template in a 20 µL SYBR-Green reaction (Meridian Bioscience, Billerica, MA, USA) with primers targeting the *ITS1* region of the fungal *rRNA* gene cluster (ITS1f: 5′-CTTGGTCATTTAGAGGAAGTAA-3′; ITS2: 5′-GCTGCGTTCTTCATCGATGC-3′). Reactions were conducted in duplicate on a Rotor-Gene Q cycler (QIAGEN, Hulsterweg, The Netherlands) with cycling conditions of 95 °C for 10 min, 45 cycles at 95 °C for 15 s, 52 °C for 20 s, and 72 °C for 60 s, which was followed by a melting curve analysis. Absolute quantification was performed using a standard curve method.

### 2.3. ITS1 Library Construction and Sequencing

To investigate the fungal taxonomic profiles in the samples, we performed *ITS1* amplicon sequencing. The amplification of this region involved fused amplification primers *ITS1*f/*ITS2* with Golay barcodes and adapter sequences (see Table S3). The PCR reactions (50 μL) were performed using the Platinum PCR SuperMix (Thermo Fisher Scientific, Waltham, MA, USA) on a S1000 Thermal Cycler (BioRad, Hercules, CA, USA) and 8 µL DNA template. After the denaturation (95 °C for 15 min), 45 cycles (95 °C for 15 s, 52 °C for 20 s, 72 °C for 60 s) were carried out, followed by a final elongation step (72 °C for 10 min). PCR products were purified using magnetic beads (NucleoMag Kit, Macherey-Nagel, Düren, Germany) and quantified on a TapeStation 2200 (Agilent Technologies, Santa Clara, CA, USA). The samples were equimolarly pooled, processed with the MiSeq Reagent Kit v2 (Illumina, San Diego, CA, USA), and sequenced on a MiSeq system (Illumina, San Diego, CA, USA) with 251 cycles.

### 2.4. ITS1 Amplicon Sequencing Analyses

Although paired-end sequencing was performed, the analysis of fungal sequences was based solely on forward reads. This decision was made for two main reasons. First, the *ITS1* region is highly variable in length and, when merging paired-end Illumina reads, some sequences may exceed the maximum allowed merged read length, which led to their exclusion, as previously reported [34]. Second, this study aimed to analyze the sequencing reads using the quality-filtering, high-resolution bioinformatics pipeline DADA2 [35]. Due to the low-quality scores of the Illumina reverse reads, only forward reads were used for downstream analysis. The QIIME2 bioinformatics software (version 2022.11) [36] was used to process the sequence data, starting with the removal of adapter sequences. Sequences quality control, denoising, dereplication, and chimera remove were performed through the DADA2 pipeline. Taxonomy assignment of the resulting amplicon sequence variants (ASVs) was carried out using a pre-trained naive Bayes classifier based on the fungal UNITE database (v9) [37]. A total of 323 fungal taxa were identified in the dataset. Taxa with a mean relative abundance greater than 1% were selected, which resulted in 17 fungal taxa that were used to generate the taxonomic composition plot. Differential abundance analysis of fungal composition at genus level was analyzed using the ‘ancombc’ R software package (v. 2.3.1). For diversity analysis of the mycobiome data, the ‘phyloseq’ R package (v. 1.45.0) was used. Shannon index was calculated to assess alpha diversity and Jaccard distances were utilized for principal coordinate analysis plots of beta diversity. The datasets (fastq files) generated in this study can be found in the SRA online repository under the accession number PRJNA1196300.

### 2.5. Antibiotic Equivalent Dose Calculation

To achieve a standardized and comparable measure of antibiotic exposure across patients, an Antibiotic Equivalent Dose (AED) score was calculated for each individual based on their cumulative antibiotic intake over a three-year observational period. The AED was determined using guideline-recommended standard daily doses for each antibiotic agent, as specified in current national cystic fibrosis treatment guidelines (AWMF 026-018) [38]. In accordance with the guidelines, the recommended daily dose (rec. daily dose) was based on patient’s age and then normalized to their individual body weight (Tables S4 and S5). The AED for each patient was determined by dividing the daily antibiotic intake (daily patient intake) by the adjusted daily dosage and multiplying the result by the duration of antibiotic therapy (treatment duration). This standardized metric enabled consistent evaluation of the potential impact of antibiotic exposure on fungal community composition in both the upper respiratory and gastrointestinal tracts.

$$\begin{array}{c} \mathrm{Antibiotic}\;\mathrm{Equivalent}\;\\ \mathrm{Dose}\;\left(\mathrm{units}\right) \end{array}=\frac{\mathrm{daily}\;\mathrm{patient}\;\mathrm{intake}\;\left(\mathrm{mg}\cdot d^{-1}\right)}{\mathrm{rec}.\;\mathrm{daily}\;\mathrm{dose}\;\left(\mathrm{mg}\cdot {\mathrm{kg}}^{-1}\right)\cdot \mathrm{patient}\;\mathrm{weigt}\;\left(\mathrm{kg}\right)}\cdot \begin{array}{c} \mathrm{treatment}\;\\ \mathrm{duration}\;\left(d\right) \end{array}$$

### 2.6. Correlation Analysis and Statistics

The R package ‘vegan’ (v. 2.6.4) was used to assess multivariate homogeneity of group dispersion, which was done through the betadispr function. Comparison of beta diversity metrics between groups was performed using the PERMANOVA statistical test. Pearson correlation analyses between fungal relative abundance and clinical parameters were analyzed with the ‘stats’ (v. 4.3.0) and ‘ggpubr’ (v. 0.6.0) R packages. Fungal co-occurrence network analysis was performed using the SparCC method, implemented in the ‘SpiecEasi’ R package (v. 1.1.3), with 100 bootstrap iterations. Network visualizations were created using Gephi software (v. 0.10.1). Inter-group comparisons were conducted using unpaired *t*-tests. Fungal taxonomic composition and comparative group visualizations were produced using GraphPad Prism (v. 9.4.1, USA). Statistical significance was defined as *p* < 0.05.

## 3. Results

Sino-nasal and stool samples from 12 CF patients were collected at three time points: before and after an acute pulmonary exacerbation and during a stable phase three months post-antibiotic treatment. For comparison, nasal lavage and stool samples were collected from healthy controls. Unless otherwise specified, the analyses of fungal community dynamics were performed using CF samples collected during the stable phase.

### 3.1. Increase Fungal Biomass and Distinct Microbiome Diversities in CF Nasal Lavage and Stool Samples Compared to Healthy Individuals

**Figure 1:**
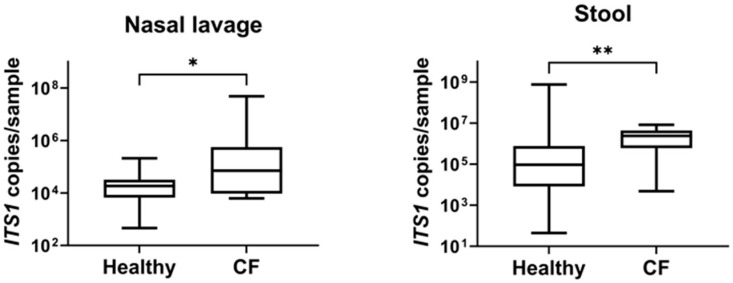
Fungal biomass in nasal lavage and stool samples during the stable phase in CF patients and healthy individuals. Box plots display quantitative analysis of fungal biomass measured by qPCR. The plots show the median number of *ITS1* copies detected. Significant *p* values: * *p* < 0.05; ** *p* < 0.01.

The fungal biomass was quantified by measuring the *ITS1* copy number for each sample. Absolute quantification of this region revealed a significantly higher fungal load in both the nasal lavage (*p* < 0.04) and stool samples (*p* < 0.001) from the CF patients compared to healthy controls (Figure 1).

**Figure 2:**
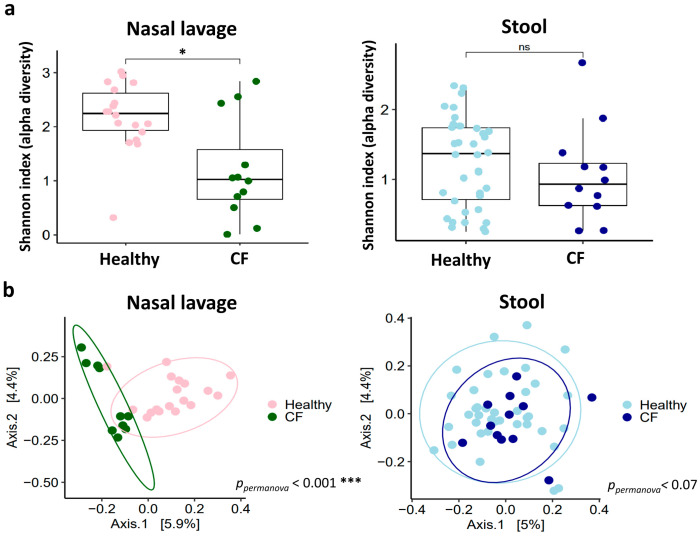
Diversity measurements of nasal lavage and stool mycobiomes in healthy individuals and CF patients during the stable phase. (**a**) Box plots of alpha diversity (Shannon index) measurements in healthy and CF nasal lavage and stool samples. *p* values were calculated using the Mann–Whitney U test for pairwise comparisons. (**b**) Principal coordinate analysis (PCoA) plot of beta-diversity based on Jaccard distances between samples. *p* values correspond to pairwise comparisons using PERMANOVA. Significant *p* values: * *p* < 0.05; *** *p* < 0.001. ns: not significant.

The fungal microbiome diversity was assessed using alpha and beta diversity metrics. Analysis of alpha diversity in the CF nasal lavage samples revealed a significantly lower Shannon index, reflecting both taxa richness and evenness, compared to that of healthy individuals (*p* < 0.01). In contrast, no significant differences in alpha diversity were observed between the stool samples of the two cohorts (*p* = 0.3) (Figure 2a). Principal coordinates analysis (PCoA) of Jaccard distances demonstrated significant differences in nasal lavage samples between the CF patients and healthy controls (*p* < 0.001). Conversely, no significant differences in beta diversity were found between the stool samples of the two groups (*p* < 0.07) (Figure 2b).

### 3.2. Significant Increased Relative Abundance of Candida spp. in CF Samples Compared to the Healthy Cohort

**Figure 3:**
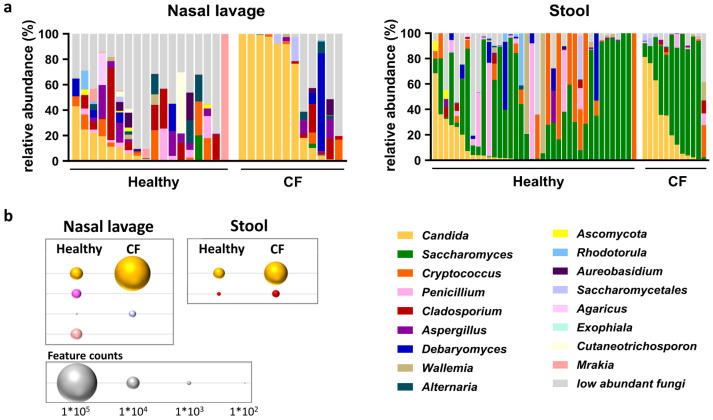
Mycobiome taxonomic analysis of nasal lavage and stool samples from healthy individuals and CF patients during the stable phase. (**a**) Shown are the relative abundances of the 17 most abundant fungal genera in samples from healthy individuals and CF patients. (**b**) Differential abundance analysis of the fungal composition measured by ANCOMBC. Bubble size represents the proportional feature counts of taxa with significant differential distribution between groups.

Analysis of the taxonomic fungal composition in nasal lavage and stool samples revealed differences in the relative abundance (RA) of several taxa between CF patients and healthy individuals (Figure 3a). In the CF nasal lavage samples, the three most abundant genera were *Candida* spp. (mean RA of 57.6%), *Debaryomyces* spp. (mean RA of 7.16%), and *Cladosporium* spp. (mean RA of 4.12%). In contrast, the healthy nasal lavage samples were characterized by *Cladosporium* spp. (mean RA of 9.26%), *Candida* spp. (mean RA of 7.97%), and *Mrakia* spp. (mean RA of 6.71%).

The stool samples from CF patients were primarily colonized by *Saccharomyces* spp. (mean RA of 56.97%), *Candida* spp. (mean RA of 27.96%), and *Cryptococcus* spp. (mean RA of 2.4%). Meanwhile, the healthy stool samples were characterized by *Saccharomyces* spp. (mean RA of 45.27%), *Cryptococcus* spp. (mean RA of 15.39%), and *Penicillium* spp. (mean RA of 7.94%).

To evaluate differences in fungal taxonomic composition, an analysis of the differential composition of microbiomes with bias correction (ANCOMBC) revealed a significantly increased RA of *Candida* spp. in both the nasal lavage and stool samples of the CF patients compared to the healthy individuals (Figure 3b). Additionally, the RA of *Saccharomycetales* spp. in the CF nasal lavage samples and *Cladosporium* spp. in the CF stool samples were significantly higher than in the healthy cohort (Table S6). In contrast, the nasal lavage samples from the healthy individuals showed a significantly increased RA of *Penicillium* and *Mrakia* spp. compared to the CF samples.

Given the significantly increased RA of *Candida* spp. in the CF patient samples, we investigated potential correlations between the presence of *Candida* spp. and key clinical factors, including cumulative antibiotic intake, long-term corticosteroid use, use of modulator therapies, lung function as measured by spirometry (FEV1%), and chronic colonization by bacterial pathogens such as *Pseudomonas aeruginosa* and *Staphylococcus aureus* in the lungs.

### 3.3. Long-Term Effect of Cumulative Antibiotic Intake Correlates with the Presence of Candida spp. in CF Nasal Lavage Samples

To accurately quantify the cumulative antibiotic exposure of each patient over a three-year period, we developed a novel and standardized metric, the Antibiotic Equivalent Dose (AED), which enables direct comparison of individuals’ antibiotic intake across different treatment regimens (Table S5). In the nasal lavage samples, Pearson correlation analysis revealed a significant positive association between the *Candida* spp. RA and cumulative antibiotic use over the three-year period, as quantified by the AED (Table 1). A significant positive correlation was also observed between the *Candida* spp. RA and long-term corticosteroid use. Additionally, the *Candida* spp. RA showed a significant negative correlation with the alpha diversity Shannon index calculated for the CF nasal lavage samples, which suggests that the observed low microbiome diversity is associated with the dominance of *Candida* spp. in these samples.

Other clinical parameters, such as modulator therapies, spirometry measurements (FEV1%), and chronic bacterial colonization by *Pseudomonas aeruginosa* and *Staphylococcus aureus* in the lungs, showed no significant correlation with the presence of *Candida* spp. in the nasal lavage samples. Similarly, none of the analyzed variables were significantly correlated with the *Candida* spp. RA in stool samples (Table S7).

### 3.4. Prolonged Antibiotic Exposure Promotes Nasal Site-Specific Candida spp. Colonization in Patients with CF, Linked to the Mode of Antibiotic Administration

**Figure 4:**
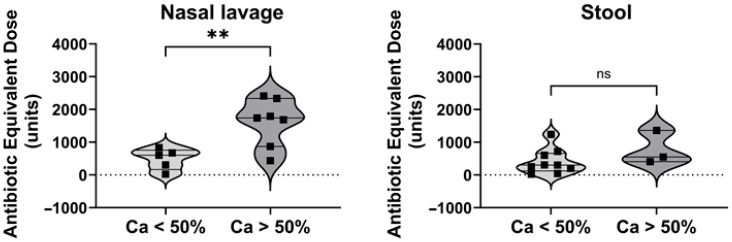
Differences in *Candida* spp. (Ca) relative abundance and cumulative antibiotic-equivalent doses in samples from Patients with CF during the stable phase. Unpaired statistical *t*-test was used for comparative analysis. ** Significant *p* values (*p* < 0.01). ns: not significant.

To address the variability in the *Candida* spp. RA across samples (Figure 3a), patients were categorized based on whether their samples showed more or less than 50% *Candida* spp. RA. Given the strong positive correlation between the *Candida* spp. RA and cumulative antibiotic intake, additional statistical analyses were performed. Patients with a high RA of *Candida* spp. (>50%) were found to have undergone significantly greater antibiotic treatment, as indicated by their Antibiotic Equivalent Dose (Figure 4), compared to the patients whose samples have less than 50% *Candida* spp. RA. Notably, this long-term effect of cumulative antibiotic intake on fungal colonization was observed exclusively in nasal lavage samples, which highlights a site-specific effect.

Furthermore, we investigated whether the mode of antibiotic administration—intravenous (iv), oral (po), or inhaled—might influence *Candida* spp. colonization at the sampled body sites. Thus, the samples were again grouped based on whether they contained more or less than 50% *Candida* spp. RA, and the mean Antibiotic Equivalent Dose for each group was compared (Figures S1 and S2). The analysis revealed that nasal lavage samples with more than 50% *Candida* spp. RA were significantly associated with patients who had received inhaled and/or intravenous antibiotic courses. In contrast, no significant differences were observed in the nasal lavage samples from patients treated with oral antibiotics. Interestingly, the route of antibiotic administration had no significant impact on the presence of *Candida* spp. in the stool samples.

### 3.5. Increased RA of Candida spp. in CF Nasal Lavage Samples After a Short Course of Antibiotics During Pulmonary Exacerbation

**Figure 5:**
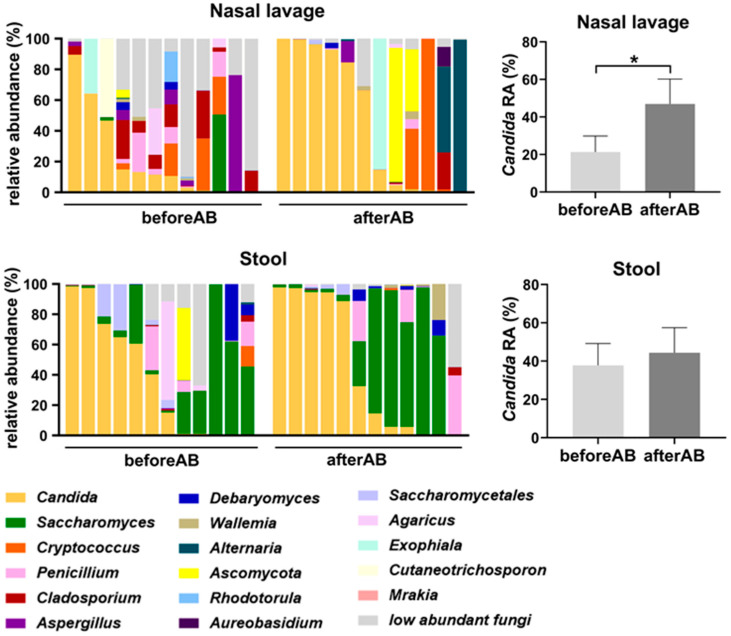
Short-term impact of antibiotic treatment on *Candida* spp. RA in nasal lavage and stool samples of patients with CF during a pulmonary exacerbation period. Fungal taxonomic summary of nasal lavage and stool samples before and after antibiotic treatment. Differences between *Candida* spp. feature counts were calculated using *t*-test (Mean ± SEM); * *p* < 0.05.

To validate the observed site-specific increase in *Candida* spp. colonization in the nasal compartment of Patients with CF and to assess the short-term impact of antibiotic treatment on the fungal community, we analyzed samples collected at two time points during the most recent pulmonary exacerbation: immediately before antibiotic initiation and after treatment. Fungal taxonomic composition analysis revealed that acute antibiotic treatment led to a significant increase in *Candida* spp. RA in nasal lavage samples (Figure 5). The mean RA of *Candida* spp. increased from 21.3% before antibiotic treatment (beforeAB) to 46.89% after the course of antibiotics was completed (afterAB). In the stool samples, no significant changes in the RA of *Candida* spp. were observed.

## 4. Discussion

This study analyzed sino-nasal lavage and stool samples from patients with cystic fibrosis (CF) and healthy controls to assess the impact of antibiotic treatments on fungal communities in the upper respiratory and gastrointestinal tracts. To investigate the long-term effects of antibiotic exposure, we recorded all antibiotic treatments over a three-year period and developed a mathematical metric termed Antibiotic Equivalent Dose (AED). Additionally, longitudinal samples from patients with CF were analyzed to assess short-term changes in the mycobiome before and after antibiotic therapy for an acute pulmonary exacerbation episode. Samples collected during the stable phase were examined for their fungal biomass, diversity, and community structure and the influence of cumulative antibiotic exposure on the mycobiome. The fungal biomass was significantly elevated in both the nasal and stool samples from the patients with CF. However, only the nasal samples exhibited reduced alpha diversity and distinct community structures. *Candida* spp. was markedly more abundant in the CF samples, particularly in the sino-nasal cavity, where the relative abundance (RA) positively correlated with the cumulative antibiotic use, especially the use of antibiotics taken via inhaled and intravenous routes. This association was specific to the nasal site, with no significant correlation being observed in the stool samples. Furthermore, the nasal samples from patients who underwent short-term antibiotic therapy during a pulmonary exacerbation showed an additional increase in *Candida* spp. RA, indicating a rapid, site-specific response. In contrast, the gut mycobiome remained stable. These findings suggest that both cumulative and localized antibiotic exposure contribute to increased fungal biomass, particularly that of *Candida* spp., in the sino-nasal mycobiome of patients with CF, while the gut mycobiome appears more resilient. This underscores the role of antibiotics in shaping fungal dynamics in CF and highlights a nasal-specific vulnerability to fungal overgrowth.

Fungal communities in the respiratory tract have long been considered transient colonizers. However, recent studies have provided evidence that the respiratory tract is permanently colonized by these microorganisms [15,39,40,41]. Fungi may enter the respiratory tract through inhalation and/or microaspiration, subsequently spreading and colonizing the airway [40]. In healthy individuals, mucociliary clearance helps maintain microbial balance, but, in individuals with compromised lung function, such as those with CF, persistent fungal colonization may occur, similar to observations in COPD and non-CF bronchiectasis respiratory diseases [42,43]. In line with this, our study demonstrated a significantly higher fungal biomass in nasal lavage samples from patients with CF during a stable phase compared to healthy controls, which suggests persistent colonization. This may be attributable to impaired sino-nasal mucociliary clearance, as previously proposed by McShane et al. [44]. During this time point, we also observed reduced alpha diversity in these samples, which is consistent with the findings of Delhaes et al., who reported decreased mycobiome diversity in CF sputum samples, which was associated with poorer clinical status [45]. A notable finding in our study was the significant enrichment of *Candida* spp. in sino-nasal and stool samples from patients with CF. In the sino-nasal samples, this enrichment was correlated with the observed reduction in fungal diversity, which indicates a possible dominance-driven dysbiosis. While *Candida* spp. are frequently reported in oral and sputum samples from patients with CF [46,47,48], their presence in the nasal cavity has rarely been documented. Wise et al. reported *Candida albicans* in 33% of sinus cultures from patients with CF [49], but broader nasal colonization has not been well-characterized. Although microaspiration from the oral cavity might explain the presence of *Candida* spp., no clinical signs of oral candidiasis were observed in our cohort.

We further investigate clinical factors that potentially influence *Candida* spp. colonization. A significant positive association was found between the *Candida* spp. abundance in nasal samples and both inhaled corticosteroid use and cumulative antibiotic exposure over three years. Corticosteroids may promote fungal persistence through immunosuppressive effects [50,51,52], but, in our study, long-term antibiotic use showed an even stronger association with *Candida* spp. overgrowth. The eradication of bacteria by antibiotics is known to provide an advantage for opportunistic fungi to colonize and proliferate, as environmental conditions, such as nutrient availability, become more favorable [53,54]. Interestingly, analysis of the route of antibiotic administration revealed that antibiotics that are given intravenously and/or inhaled were significantly associated with the nasal lavage samples showing a high RA of *Candida* spp. In line with these results, Noni et al. previously proposed that the chronic colonization of *Candida* spp. in the lung might be associated with the duration of inhaled antibiotics treatments [30]. In our study, the novel calculation of cumulative inhaled antibiotic exposure over three years revealed that not just the therapy duration, but the total dosage, might contribute to fungal overgrowth. This effect was not limited to chronic exposure, acute antibiotic treatment during pulmonary exacerbations also led to significant increases in *Candida* spp. abundance in sino-nasal samples. Given the rich vascularization of the nasal mucosa, systemic antibiotics may contribute to local fungal colonization by reaching this site via circulation [55]. This systemic exposure can disrupt commensal bacterial populations within the nasal cavity, reducing bacterial–fungal competition and allowing fungal species, such as *Candida* spp., to proliferate. These findings support the hypothesis that the upper airway, particularly the nasal cavity, may act as a reservoir for fungal colonization capable of seeding the lower respiratory tract [56]. Moreover, the prolonged half-life and tissue retention of certain systemic antibiotics may enhance this effect by extending the duration of bacterial dysbiosis, thereby further facilitating fungal overgrowth [57,58]. Antibiotic use can also indirectly impair antifungal immunity by altering microbiota-driven immune signaling, potentially weakening mucosal defenses against fungal colonization [59]. Additionally, inhaled antibiotics achieve high local concentrations primarily in the airways while having minimal systemic absorption [60]. As a result, they might selectively perturb the airway microbiota, with a more localized impact on bacterial communities and subsequently on fungal overgrowth. Overall, both the route of administration and cumulative antibiotic exposure appear to play critical roles in the risk of fungal colonization. Persistent colonization by *Candida* spp. could thus have downstream implications for lung health in patients with CF.

While the pathogenic role of *Candida* spp. in CF remains a subject of ongoing research, emerging evidence suggests it may not be benign but could actively contribute to disease progression. For instance, *Candida* spp. can form biofilms and enhance microbial persistence and resistance to antimicrobial treatments [11,61]. Moreover, synergistic interactions with pathogens such as *Pseudomonas aeruginosa* may enhance virulence and contribute to airway inflammation [62,63,64]. In addition, cell wall components like *Candida* β-glucans can trigger strong immune responses [65,66], potentially worsening the already elevated basal inflammatory state of CF airways. Moreover, colonization of the nasal cavity by *Candida* spp. may impair mucociliary clearance by disrupting the epithelial integrity or altering mucus properties, thus facilitating both fungal persistence and secondary bacterial infections. Although we did not observe a direct correlation between nasal *Candida* spp. abundance and lung function in this study, the strong association with antibiotic exposure and its rapid increase following treatment highlight its clinical relevance. These findings suggest that sino-nasal *Candida* spp. overgrowth may not only reflect microbial dysbiosis but also represent a potential preclinical marker or risk factor for exacerbations and disease progression. Future longitudinal studies are warranted to determine whether nasal *Candida* colonization might predict clinical outcomes in CF and whether it could represent a target for antifungal or adjunctive therapies.

Fungi are common colonizers of the healthy human gut, where they contribute to maintaining homeostasis [67]. Analysis of the fungal load in CF stool samples showed increased fungal biomass as compared to that in stool samples of healthy individuals. These results may be explained by CFTR dysfunction in patients with CF, which leads to frequent antibiotic treatments, hyperacidity due to a lack of pancreatic bicarbonate, maldigestion, CF-related diabetes, or hepatopathy, all of which contribute to intestinal dysbiosis [68]. In contrast, the overall lower fungal biomass observed in the healthy samples may help explain the apparent dominance of individual fungal genera in some of these cases. For example, certain healthy individuals showed a high relative abundance of *Saccharomyces* spp., *Penicillium* spp., or *Cryptococcus* spp. in their stool samples. Similar patterns have been reported previously, which suggests that low fungal biomass or limited recovered material from healthy individuals can result in the dominance of single taxa [69,70]. No significant differences in fungal diversity were observed; however, two dominant taxa were identified: *Saccharomyces* spp. and *Candida* spp., with the latter being significantly enriched in the CF samples. Interestingly, none of the clinical variables tested, including long-term Antibiotic Equivalent Dose scores, correlated with the elevated *Candida* spp. abundance in these patients. Various factors such as diet, body weight, and geographic location influence the gut fungal composition [71,72]. While we lack dietary data from our cohort, dietary factors may explain the prominence of these fungi in these samples. Interestingly, we observed a possible antagonistic behavior between *Saccharomyces* spp. and *Candida* spp.; samples with a high RA of *Saccharomyces* spp. showed a lower RA of *Candida* spp. and vice versa. This aligns with in vitro findings by Krasowska et al., who showed that *Saccharomyces boulardii* can inhibit *Candida albicans* virulence and biofilm formation [73]. To further explore fungal interactions, we performed Pearson correlation analyses and visualized them via network diagrams (Figure S3). Interestingly, the patients with CF showed more significant correlations involving *Candida* spp. than healthy controls (Table S8). In sino-nasal lavage samples, *Candida* spp. negatively correlated with *Alternaria* and *Cladosporium* spp., while, in the gut, the already observed antagonistic behavior between *Candida* spp. and *Saccharomyces* spp. was statistically significant. Additional distinct fungal correlations were observed in both body sites and cohorts, which underscores the complexity of the fungal ecosystem. However, further studies are needed to better understand the fungal cross-talk in health and disease.

Our study has several limitations, including a relatively heterogeneous patient age range, including both pediatric and adult patients with CF. While age can influence individuals’ microbiome composition [74], its effect could not be distinguished from that of long-term antibiotic exposure in our statistical analysis. As a result, age was not included in the statistical modeling. Another limitation is the relatively small sample size, which may impact the statistical power of this study and the detectable effect sizes. Consequently, smaller effects might remain unnoticed due to the high interindividual variation in the mycobiome. Further studies with larger sample sizes are needed to validate the findings observed in our cohort. Additionally, most samples were collected before the widespread introduction of the highly effective triple-combination therapy elexacaftor/tezacaftor/ivacaftor (ETI). This is relevant, as ETI has demonstrated substantial clinical benefits and significant impacts on both the bacterial microbiome [75,76] and airway inflammation [77]. Although no correlation was observed between the modulator therapies and the relative amount of *Candida* spp. found in the samples of this study, the predominance of patients treated prior to the ETI era may be considered a strength, as it provides a valuable baseline for understanding fungal colonization in the absence of highly effective modulation. In the present patient cohort, a direct comparison between individuals with and without prior antibiotic treatments was not possible. However, recent studies have shown that ETI markedly reduces annual pulmonary exacerbation rates and, consequently, the need for supportive chronic therapies, such as antibiotics [78,79]. In line with this, future studies involving larger, age-stratified CF cohorts on highly effective triple-combination therapy (HEMT), that directly compare patients with and without long-term antibiotic exposure, are warranted to validate and expand these findings.

## 5. Conclusions

To our knowledge, this study is the first to systematically evaluate both the long- and short-term effects of antibiotics on fungal colonization in patients with CF using a custom-developed standardized metric, the Antibiotic Equivalent Dose. We observed significant overgrowth of *Candida* spp. in both the sino-nasal cavity and gut. Notably, nasal *Candida* overgrowth was strongly linked to cumulative antibiotic exposure over three years and further increased after the antibiotic therapy during exacerbations, which indicates a site-specific, antibiotic-driven dysbiosis. This finding raises the possibility that fungal overgrowth could extend to other respiratory sites and contribute to worsening CF pathology. Our results highlight the importance of further investigating the role of fungi, especially *Candida* spp., in CF disease development and progression, emphasizing the need to consider fungal overgrowth as a potential factor in airway pathology and to support ongoing research into its clinical significance and therapeutic potential.

## Figures and Tables

**Table 1:** **Pearson** correlation analysis between *Candida* RA present in nasal lavage samples of CF patients and clinical variables.

Nasal Lavage Samples			*Candida* spp. RA
	**Antibiotic Equivalent Dose** **(over 3 years)**	Correlation coefficient	0.674
		Sig. (2-tailed)	0.016 *
		N	12
	**Corticoids (over 3 years)**	Correlation coefficient	0.620
		Sig. (2-tailed)	0.031 *
		N	12
	**Modulator (double/single)**	Correlation coefficient	0.230
		Sig. (2-tailed)	0.472
		N	12
	**FEV1%**	Correlation coefficient	0.280
		Sig. (2-tailed)	0.404
		N	11
	**Shannon index**	Correlation coefficient	−0.743
		Sig. (2-tailed)	0.006 **
		N	12
	***P. aeruginosa*** **chronic lower airway colonization**	Correlation coefficient	0.285
		Sig. (2-tailed)	0.369
		N	12
	***S. aureus*** **chronic lower airway colonization**	Correlation coefficient	0.275
		Sig. (2-tailed)	0.388
		N	12

** Correlation is significant at the 0.01 level (two-tailed). * Correlation is significant at the 0.05 level (two-tailed). Modulator: double (ivacaftor/lumacaftor or ivacaftor/tezacaftor); single (ivacaftor).

## Data Availability

The datasets (fastq files) generated in this study can be found in the SRA online repository under the accession number: PRJNA1196300.

## Supplementary Materials

The following supporting information can be downloaded at https://www.mdpi.com/article/10.3390/jof11090631/s1, Figure S1. Comparative analysis of cumulative antibiotic equivalent doses received by CF patients, based on the relative abundance of *Candida* (Ca) in their nasal lavage samples. Figure S2. Comparative analysis between *Candida* relative abundance (Ca) and antibiotic equivalent doses of CF stool samples. Figure S3: Network visualization of Pearson Correlation Coefficients (PCC) among fungal taxa in nasal lavage and stool samples from CF and healthy subjects. Table S1. Clinical data from cystic fibrosis patients. Table S2. Cohort description. Table S3. Primer constructs for sequencing library preparation. Table S4: Recommended daily dosages of antibiotics based on established guidelines (AWMF 026-018). Table S5. CF patients course of antibiotic therapies and calculated antibiotics equivalent dose over a 3-year period. TableS6. Differentially abundant bacterial taxa between healthy and CF nasal lavage and stool samples identified by ANCOMBC. Table S7. Pearson Ccorrelation analysis between *Candida* relative abundance (RA) found in the stool samples of CF patients and clinical parameters. Table S8: Significant fungal associations identified in CF and healthy control nasal lavage and stool samples. References [38,80,81,82,83,84,85,86,87,88,89,90,91,92,93,94,95,96] are cited in the Supplementary Materials.

## Abbreviations

The following abbreviations are used in this manuscript:

CF	cystic fibrosis
AED	Antibiotic Equivalent Dose
CFTR	CF transmembrane conductance regulator
spp.	several species
BMI	body mass index
FEV1%	Forced expiratory volume in one second as a percentage
rec.	recommended
d	days
ASVs	amplicon sequence variants
PCoA	principal coordinates analysis
RA	relative abundance
iv	intravenous
po	per os
beforeAB	before antibiotic treatment
afterAB	after antibiotic treatment
HEMT	Highly Effective Triple-Combination Therapy
ETI	elexacaftor/tezacaftor/ivacaftor
