# Supplementary material for: Impact of Antibiotic Therapy on the Upper Respiratory Tract and Gut Mycobiome in Patients with Cystic Fibrosis
Source: J Fungi (Basel). 2025 Aug 28;11(9):631. doi: 10.3390/jof11090631 (PMC12471002; doi:10.3390/jof11090631)
Supplement: Supplementary file 1 [file jof-11-00631-s001.zip › jof-3739452-supplementary.pdf]

Impact of Antibiotic Therapy on the Upper Respiratory Tract and Gut Mycobiome in Patients with Cystic Fibrosis

Cristina Zubiria-Barrera [1,2,3,*], Malena Bos [1,2,3], Robert Neubert [1,2,3], Jenny Fiebig [1,2], Michael Lorenz [4], Michael Hartmann [5], Jochen G. Mainz [6], Hortense Slevogt [1,2,3,†] and Tilman E. Klassert [1,2,3,†]

1 Department of Respiratory Medicine and Infectious Diseases, Medical School Hannover (MHH), German Center for Lung Research (DZL), Research Network "BREATH", 30625 Hannover, Germany; malena.bos@helmholtz-hzi.de (M.B.); robert.neubert@helmholtz-hzi.de (R.N.); fiebig.jenny@mh-hannover.de (J.F.); slevogt.hortense@mh-hannover.de (H.S.); tilman.klassert@helmholtz-hzi.de (T.E.K.)
2 Dynamics of Respiratory Infections, Helmholtz Centre for Infection Research, 38124 Braunschweig, Germany
3 Cluster of Excellence RESIST (EXC 2155), Hannover Medical School, Carl-Neuberg-Straße 1, 30625 Hannover, Germany
4 Department of Pediatric Pulmonology/Allergology, Section Cystic Fibrosis Centre for Children and Adults, University Hospital Jena, 07747 Jena, Germany; michael.lorenz@med.uni-jena.de
5 Hospital Pharmacy, University Hospital Jena, 07747 Jena, Germany; michael.hartmann@med.uni-jena.de
6 Cystic Fibrosis Center, Klinikum Westbrandenburg, Brandenburg Medical School (MHB) University, 16816 Neuruppin, Germany; j.mainz@uk-brandenburg.de
* Correspondence: cristina.zubiriabarrera@helmholtz-hzi.de
† These authors contributed equally to this work.

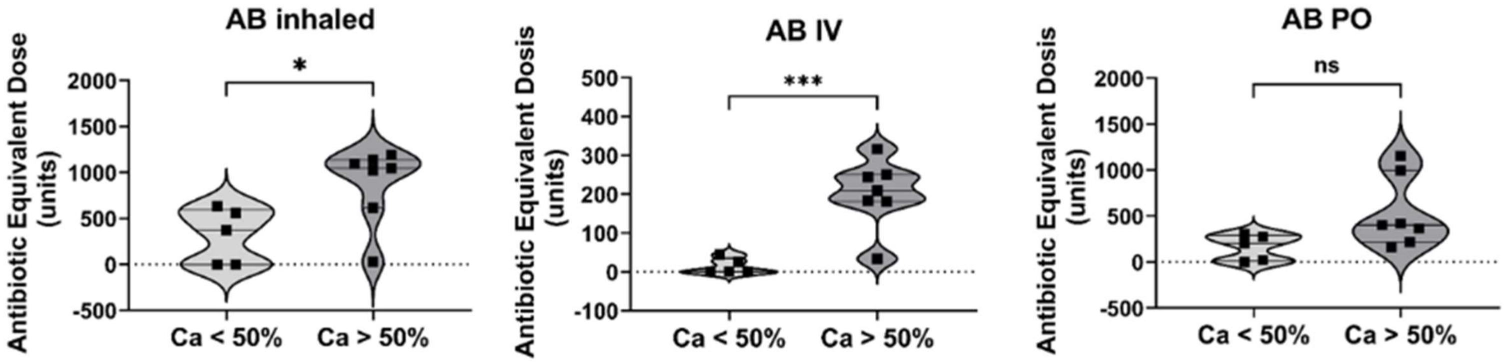

Figure S1. Comparative analysis of cumulative antibiotic equivalent doses received by CF patients, based on the relative abundance of *Candida* (Ca) in their nasal lavage samples. Antibiotic equivalent doses were calculated according to the antibiotics received by the patients, categorized by route of administration: inhalation, intravenous (IV), or oral (PO). Patients were grouped by *Candida* relative abundance (RA) in nasal lavage samples: <50% vs. ≥50% RA. Statistical comparisons between groups were conducted using an unpaired t-test. * indicates statistically significant differences ($p < 0.05$). ns: not significant.

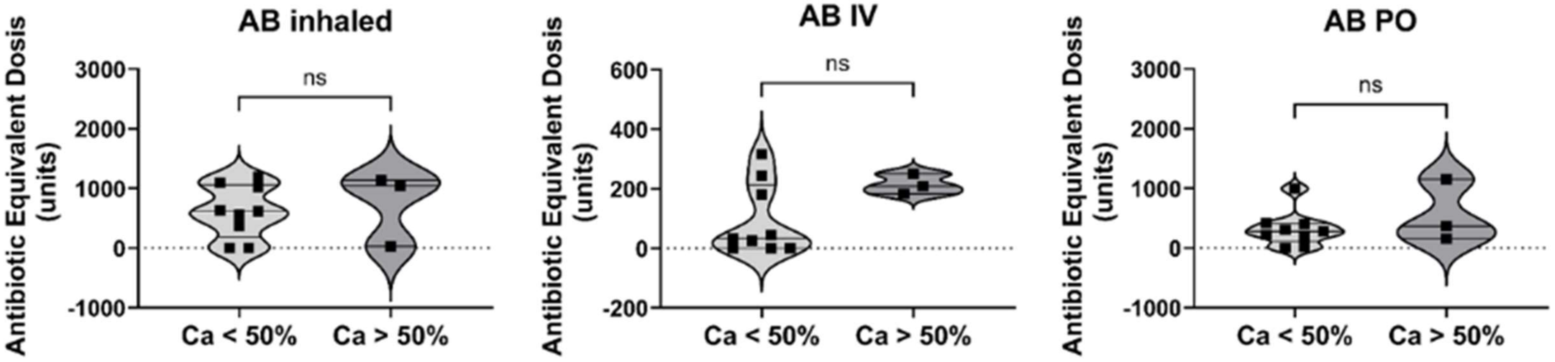

Figure S2. Comparative analysis between *Candida* relative abundance (Ca) and antibiotic equivalent doses of CF stool samples. Routes of antibiotics administration was considered for antibiotic equivalent dose determination. Unpaired statistical t-test was used for group comparison. ns: not significant.

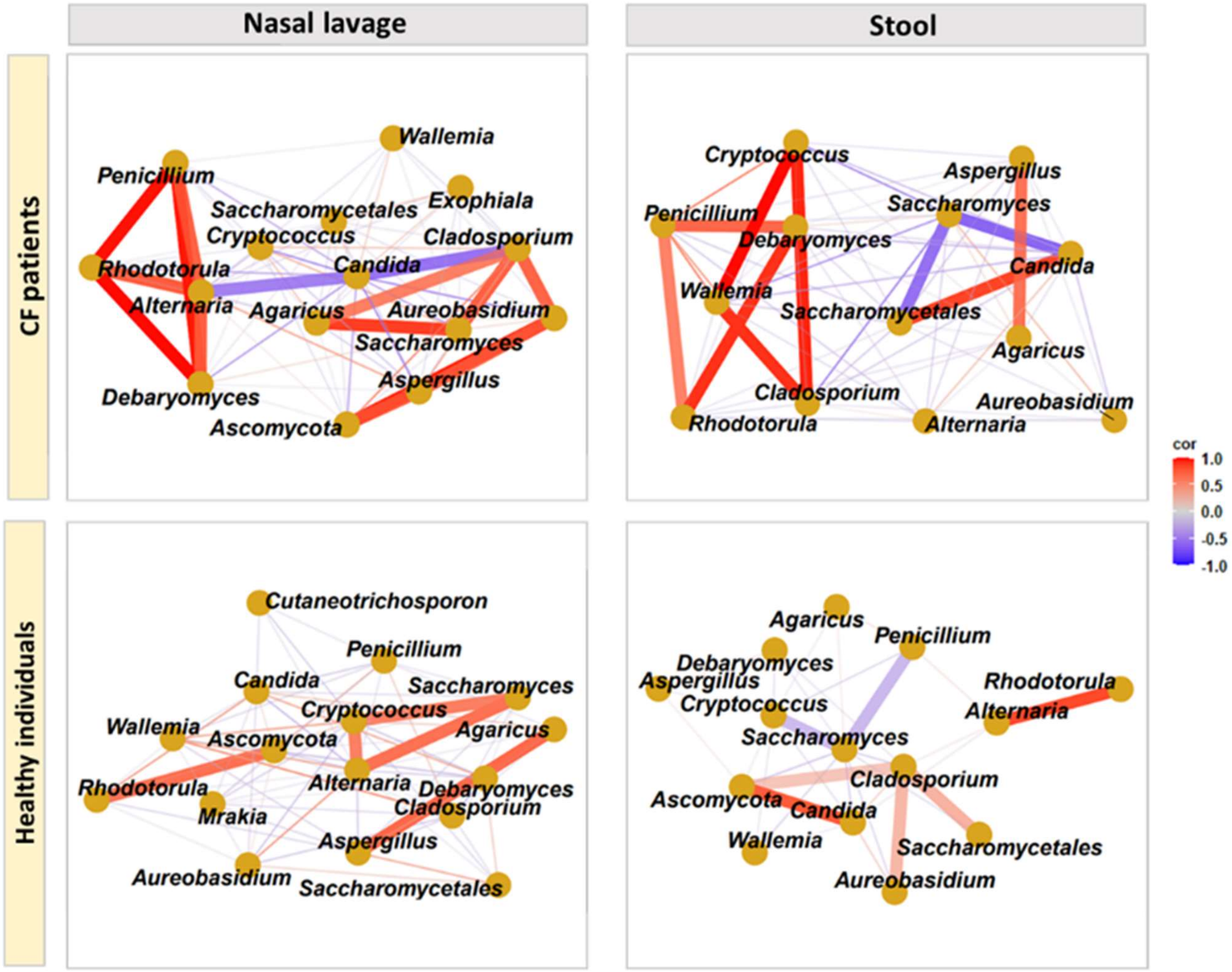

Figure S3: Network visualization of Pearson Correlation Coefficients (PCC) among fungal taxa in nasal lavage and stool samples from CF and healthy subjects. The network diagram illustrates Pearson correlation coefficients (PCC > 0.1) for main fungal taxa in samples from both CF patients and healthy controls. Positive PCCs are depicted in red, while negative PCCs are shown in blue. Significant positive and negative associations are represented by thick lines, while non-significant associations are shown with thin lines. A positive fungal association suggests a higher likelihood that the two fungi co-localize within the same sample, meaning they tend to be present together. In contrast, a negative correlation indicates that the presence of one fungus is associated with the absence of the other, suggesting they are less likely to co-localize.

Table S1. Clinical data from cystic fibrosis patients

Patient	Gender	Age	BMI	FEV1%	CFTR mutation	Corticoids	Modulator	*P. aeruginosa** colonization	*S. aureus** colonization
**P1**	male	37	27.1	76	F508del/F508del	yes	double	yes	yes
**P2**	male	33	21.5	95.3	F508del/405+1G-A	no	NA	yes	no
**P3**	female	41	20.5	81.4	F508del/2789+5G-A	yes	double	yes	yes
**P4**	female	39	27.2	123	F508del/2789+5G-A	yes	NA	yes	yes
**P5**	male	34	18.6	40	F508del/F508del	yes	double	yes	yes
**P6**	male	38	19.1	121	F508del/G551D	no	single	no	yes
**P7**	male	9	15.3	97.1	F508del/F508del	yes	double	no	yes
**P8**	male	4	15.5	NA	F508del/N1303K	no	NA	no	yes
**P9**	female	32	18.2	95.3	F508del/F508del	yes	double	no	yes
**P10**	female	6	16.9	83.5	F508del/F508del	yes	double	no	yes
**P11**	male	22	17.6	37.9	F508del/F508del	yes	double	yes	no
**P12**	female	11	15	82.7	F508del/F508del	yes	double	yes	yes

* diagnosed from sputum, nasal lavage or throat swabs samples

Modulator: double (Ivacaftor/Lumacaftor or Ivacaftor/Tezacaftor); single (Ivacaftor); NA: not applicable

Ivacaftor = Kalydeco, used for gating mutations, primarily Class III mutations; e.g: G551D

Ivacaftor/Lumacaftor = Orkambi, used for patients homozygous for the F508del mutation

Ivacaftor/Tezacaftor = Symkevi, used for F508del, either: homozygous F508del or one F508del + another responsive mutation

Table S2. Cohort description

	**Healthy subjects (n = 38)**	**Cystic fibrosis patients (n= 12)**
Samples collected:		
Nasal lavage	18	12
Stool	38	12
Age (years)	28 (20 - 35)	32.5 (9.5 - 37.75)
Gender (male/female)	15 / 23	7 / 5

Table S3. Primer constructs for sequencing library preparation

Forward construct	Components	Sequence
	5′ - Illumina Adapter	AATGATACGGCGACCACCGAGATCTACAC
	Forward linker	GG
	Forward primer (ITS1f)	CTTGGTCATTTAGAGGAAGTAA
**Reverse construct**	**Components**	**Sequence**
	Reverse complement 3′ - Illumina Adapter	CAAGCAGAAGACGGCATACGAGAT
	Golay barcode	NNNNNNNNNNNN
	Reverse linker	CG
	Reverse primer (ITS2)	GCTGCGTTCTTCATCGATGC

Table S4: Recommended daily dosages of antibiotics based on established guidelines (AWMF 026-018)

Antibiotic	Pharm. form	Daily dosage	Dosage-interval (h)	Daily pharm. freq.	Therapy duration	Ref.
**β-lactams**						
**Monobactams**						
Aztreonam	i.v.	150 mg/kg/d	6	4x	14 d	[1]
	inhaled	75 mg	8	3x	4 w	[1]
**Penicillins**						
AmoClav 875 mg/125 mg (amoxicillin/clavulanic acid)	p.o.	≥ 40 kg ≙ 2x 875/125 mg<br>25 < 40 kg ≙ 2x 22.5/3.2 mg/kg	12	2x	14 d (CF pat. ~ 20d.)	[2]
AmoClav Liquid 125 mg/31,25 mg per 5 ml (amoxicillin/clavulanic acid)	p.o.	20 mg/5 mg - 40 mg/10 mg per kg/day in 3 doses	8	3x	14 d (CF pat. ~ 20d.)	[3]
AmoClav Liquid 400 mg/57 mg per 5 ml (amoxicillin/clavulanic acid)	p.o.	25 mg/3.6 mg - 45 mg/6.4 mg per kg/day in 2 doses	12	3x	14 d (CF pat. ~ 20d.)	[4]
Infectomox liquid 750 mg per 5 mL (amoxicillin)	p.o.	Children: 40 - 90 mg/kg/d<br>5 - 7.5 kg ǀ 3 - 6 months ǀ 300 - 675 mg/d<br>7.6 - 10 kg ǀ 6 - 12 months ǀ 400 - 900 mg/d<br>11 - 15 kg ǀ 1 - 3 years ǀ 600 - 1350 mg/d<br>16 - 22.5 kg ǀ 3 - 6 years ǀ 900 - 2000 mg/d<br>23 - 30 kg ǀ 6 - 10 years ǀ 1200 - 2700 mg/d<br>31 - 40 ǀ 10 - 12 years ǀ 3000 mg/d	4 - 12	2 - 4x	20 d	[5]
Piperacillin/tazobactam	i.v.	240 - 400 mg/kg	8	3x	14 d	[1]
Ticarcillin/clavulanic acid	i.v.	500 - 750 mg/kg/d	6	4x	14 d	[1]
**Cephalosporins**						
Ceftazidime	i.v.	150 - 250 mg/kg<br>or<br>150 mg/kg/d continuous infusion	6 - 8<br>or<br>continuous	3 - 4x	14 d	[1]
Cefepime	i.v.	100 - 150 mg/kg/d	6 - 8		14 d	[1]
Cefpodoxime (200 mg)	p.o.	≥ 12 years: 2x 200 mg	12	2x	5 - 10 d (CF pat. ~ 20d.)	[6]

Antibiotic	Pharm. form	Daily dosage	Dosage-interval (h)	Daily pharm. freq.	Therapy duration	Ref.
**Cephalosporins**						
Cefpodoxime liquid<br>40 mg per 5 mL	p.o.	5 - 9.9 kg ǀ 2x 20 mg<br>10 - 14.9 kg ǀ 2x 40 mg<br>15 - 19.9 kg ǀ 2x 60 mg<br>20 - 24.9 kg ǀ 2x 80 mg<br>≥ 25 kg ǀ 2x 100 mg	12	2x	5 - 10 d (CF pat. ~ 20d.)	[7]
Cefuroxime	p.o.	≥ 40 kg ≙ 2x 500 mg<br>< 40 kg ≙ 2x max. 250 mg	12	2x	5 - 10 d (CF pat. ~ 20d.)	[6]
**β-lactams**						
**Carbapenems**						
Imipenem	i.v.	50-100 mg/kg/d	6	4x	14 d	[1]
Meropenem	i.v.	60 - 120 mg/kg	8	3x	14 d	[1]
**Aminoglycosides**						
Amikacin	i.v.	30 mg/kg/d **	24	1x	14 d	[1]
Tobramycin	i.v.<br>inhaled	10 mg/kg **<br>160 - 600 mg	24<br>12	1x<br>2x	14 d<br>4 w	[1, 8]
**Quinolones**						
Ciprofloxacin	p.o.<br>i.v.	30 mg/kg/d in 2 doses<br>30 mg/kg/d	12<br>8	2x<br>3x	3 - 12 w	[1]
**Polymyxins**						
Colistin (polymyxin E)	i.v.<br>inhaled	2.5 - 5 mg/kg/d ǀ 75.000 IU/kg/d<br>(480 mg/d ǀ 6 Mio IU)<br>2 - 6 Million IU<br>(1 Mio IU = 80 mg colistimethate sodium)	8<br>12	3x<br>2x	3 - 12 w	[1, 8]
**Macrolides**						
Azithromycin	p.o.	≥ 45 kg ≙ 1x 500 or 1000mg	24 (72)	1x<br>(3x/week as prophylaxis)	1-3 d / long term	[6]

Antibiotic	Pharm. form	Daily dosage	Dosage-interval (h)	Daily pharm. freq.	Therapy duration	Ref.
**Macrolides**						
Azithromycin Liquid<br>(200 mg per 5 mL)	p.o	15 - 25,9 kg ǀ 1x 200 mg<br>26 - 35,9 kg ǀ 1x 300 mg<br>36 - 45 kg ǀ 1x 400 mg	24	1x	3-5 d	[9]
Erythromycin<br>(200-600 mg per 5 mL)	p.o.	Children: 25 - 40 mg/kg/d in 2 doses<br>< 3.5 kg ǀ 2x 50 mg<br>3.5 - 7.5 kg ǀ 2x 100 mg<br>7.5 - 12.5 kg ǀ 2x 200 mg<br>12.5 - 17.5 kg ǀ 2x 300 mg<br>17.5 -25 kg ǀ 2x 400 mg<br>25 - 35 ǀ 2x 600 mg	12	2x	20 d	[10-12]
Clarithromycin<br>(125-250 mg per 5 mL)	p.o.	Children ≤ 12 years: 15 mg/kg/d in 2 doses<br>8 - 11 kg ǀ 1 -2 years ǀ 2x 62,5 mg<br>12 - 19 kg ǀ 2 - 4 years ǀ 2x 125 mg<br>20 - 29 kg ǀ 4 - 8 years ǀ 2x 187.5 mg<br>30 - 40 ǀ 8 - 12 years ǀ 2x 250 mg	12	2x	(5 - 10 d for children)<br>(6 - 14 d for adults and young people)<br>CF mostly 20 d	[13, 14]
**Sulfonamides**						
Co-trimoxazole 800/160<br>(sulfamethoxazole/trimethoprim)	p.o.	6 - 12 years 400/80 mg<br>> 13 years 800/160 mg	12	2x	5 - 8 d (CF pat. ~ 20 d)	[15]
Co-trimoxazole Liquid<br>(200/40 mg per 5 mL)<br>(400/80 mg per 5 mL)	p.o.	6 weeks - 5 months 100/20 mg<br>6 months - 5 years 200/40 mg<br>6 years - 12 years 400/80 mg<br>> 13 years 800/160 mg	12	2x	> 5 d	[16, 17]
**Tetracyclines**						
Doxycycline	p.o.	> 70 kg ≙ 200 mg permanent<br>45 kg - 70 kg ≙ 200 mg at 1st day<br>100 mg from 2nd day<br>< 45 kg ≙ 4.4 mg/kg BWT at 1st day<br>2.2 mg/kg BWT from 2nd day	24	1x	7 - 21 d	[18]

pharm. = pharmaceutical; freq. = frequency; h = hours; d = days; w = weeks; BWT = Body weight; SD = single dose
i.v. = intravenously; p.o. = *per os* (orally)
** drug plasma level required

Table S5. CF patients course of antibiotic therapies and calculated antibiotics equivalent dose over a 3-year period

Patient	Antibiotic inhaled	Antibiotic *per os* (PO)	Antibiotic intravenously (IV)	AB Equivalent Dose Inhaled (units)	AB Equivalent Dose PO (units)	AB Equivalent Dose IV (units)
**P1**	Colistin<br>Tobramycin	Cefuroxime<br>Doxycycline<br>Azithromycin	Tobramycin<br>Meropenem<br>Colistin	1137.5	365.72	183.15
**P2**	Levofloxacin	Ceftazidime<br>Amoxicillin-clavulanic acid<br>Ciprofloxacin<br>Doxycycline<br>Azithromycin	Meropenem	1190.5	419.41	181.28
**P3**	Colistin<br>Tobramycin	Azithromycin	Meropenem	1046.5	1152.63	209.31
**P4**	Colistin	-	Tobramycin<br>Piperacillin-Tazobactam<br>Meropenem	559	0	44.57
**P5**	Colistin<br>Aztreonam	Trimethoprim-Sulfamethoxazole<br>Ciprofloxacin<br>Azithromycin	Tobramycin<br>Ceftazidime<br>Meropenem<br>Fosfomycin	1095	994.82	244.27
**P6**	-	Amoxicillin-clavulanic acid<br>Cefuroxime	-	0	21.36	0
**P7**	Colistin<br>Tobramycin	Amoxicillin-clavulanic acid<br>Trimethoprim-Sulfamethoxazole<br>Cefuroxime<br>Ciprofloxacin	Tobramycin<br>Ceftazidime	616	217.33	33.97
**P8**	-	Amoxicillin-clavulanic acid<br>Trimethoprim-Sulfamethoxazole<br>Cefaclor	-	0	304.37	0
**P9**	Levofloxacin	Amoxicillin-clavulanic acid<br>Trimethoprim-Sulfamethoxazole<br>Doxycycline	Tobramycin<br>Ceftazidime<br>Piperacillin-Tazobactam<br>Meropenem	28	157.7	250.27
**P10**	Tobramycin	Amoxicillin-clavulanic acid<br>Trimethoprim-Sulfamethoxazole<br>Cefuroxime<br>Ciprofloxacin<br>Erythromycin	Tobramycin<br>Ceftazidime<br>Linezolid	372	277.01	25.84
**P11**	Colistin<br>Tobramycin	Trimethoprim-Sulfamethoxazole<br>Azithromycin	Tobramycin<br>Piperacillin-Tazobactam<br>Meropenem	1021.5	402	0
**P12**	Tobramycin	Amoxicillin-clavulanic acid<br>Trimethoprim-Sulfamethoxazole<br>Cefuroxime<br>Ciprofloxacin	-	633	200.98	0

TableS6. Differentially abundant bacterial taxa between healthy and CF nasal lavage and stool samples identified by ANCOMBC

Healthy vs. CF	Taxa	*p adj*. value ($p < 0.05$)	Healthy (FC)	CF (FC)	RA %_Healthy	RA %_CF
**Nasal lavage**	*Candida* spp.	1.33×10^{-4}	9997.7	78560.5	7.97	57.55
	Penicillium spp.	7.59×10^{-4}	5518.1	15.3	3.86	0.04
	Saccharomycetales spp.	0.019	229.4	3017.8	0.23	2.21
	Mrakia spp.	6.68×10^{-24}	8054.3	0.0	6.71	0
**Stool**	*Candida* spp.	2.00×10^{-07}	7403.9	32686.3	6.43	26.96
	Cladosporium spp.	6.95×10^{-06}	815.6	3128.8	0.83	1.43

FC = feature counts; RA = relative abundance

Table S7. Pearson **correlation analysis between *Candida* relative abundance (RA) found in the stool samples of CF patients and clinical parameters**.

Stool samples			*Candida* spp. RA
	**Antibiotic Equivalent Dose (over 3 years)**	Correlation coefficient	0.015
		Sig. (2-tailed)	0.962
		N	12
	**Corticoids (over 3 years)**	Correlation coefficient	0.178
		p- value	0.581
		N	12
	**Modulator (double / single)**	Correlation coefficient	0.386
		p- value	0.215
		N	12
	**FEV1 %**	Correlation coefficient	0.044
		p- value	0.897
		N	11
	**Shannon index**	Correlation coefficient	0.139
		p- value	0.666
		N	12
	***P. aeruginosa* colonization**	Correlation coefficient	0.058
		Sig. (2-tailed)	0.859
		N	12
	***S. aureus* colonization**	Correlation coefficient	0.363
		Sig. (2-tailed)	0 .247
		N	12

** Correlation is significant at the 0.01 level (2-tailed).
 * Correlation is significant at the 0.05 level (2-tailed).

Table S8: Significant fungal associations identified in CF and healthy control nasal lavage and stool samples

Groups	Significant fungal associations	Pearson correlation coefficient (PCC)	*p*-value (< 0.05)
**CF** *Nasal lavage*	*Debaryomyces - Rhodotorula*	0.99	2.91×10^{-11}
	Penicillium - Rhodotorula	0.98	4.08×10^{-08}
	Penicillium - Debaryomyces	0.97	9.91×10^{-08}
	Ascomycota - Aureobasidium	0.95	0.00000171
	Saccharomyces - Agaricus	0.88	0.000144
	Aspergillus - Ascomycota	0.78	0.00284
	Alternaria - Rhodotorula	0.78	0.00277
	Debaryomyces - Alternaria	0.77	0.00355
	Saccharomyces - Cladosporium	0.76	0.0041
	Penicillium - Alternaria	0.76	0.00383
	Cladosporium - Aureobasidium	0.75	0.00472
	Aspergillus - Aureobasidium	0.72	0.00784
	Cladosporium - Agaricus	0.65	0.0233
	Candida *- Alternaria*	-0.58	0.0482
	Candida *- Cladosporium*	-0.63	0.0272
**CF** *Stool*	*Cryptococcus - Wallemia*	0.99	1.99×10^{-09}
	Cryptococcus - Cladosporium	0.95	0.00000329
	Cladosporium - Wallemia	0.92	0.0000297
	Debaryomyces - Rhodotorula	0.92	0.0000301
	Candida *- Saccharomycetales*	0.86	0.000285
	Penicillium - Debaryomyces	0.74	0.0058
	Aspergillus - Agaricus	0.7	0.0118
	Penicillium - Rhodotorula	0.64	0.0249
	Saccharomyces - Saccharomycetales	-0.64	0.025
	Candida *- Saccharomyces*	-0.68	0.0154
**Healthy controls** *Nasal lavage*	*Aspergillus - Agaricus*	0.68	0.00183
	Ascomycota - Rhodotorula	0.64	0.00398
	Saccharomyces - Alternaria	0.63	0.00506
	Saccharomyces - Cryptococcus	0.58	0.0125
	Cryptococcus - Alternaria	0.57	0.0126
**Healthy controls** *Stool*	*Alternaria - Rhodotorula*	0.83	7.76×10^{-11}
	Candida *- Ascomycota*	0.76	0.000000023
	Cladosporium - Saccharomycetales	0.41	0.00969
	Cladosporium - Aureobasidium	0.39	0.0152
	Cladosporium - Ascomycota	0.33	0.0429
	Saccharomyces - Cryptococcus	-0.34	0.0388
	Saccharomyces - Penicillium	-0.35	0.0289

blue: negative correlation; red: positive correlation

References

1. Schwarz C, Schulte-Hubbert B, Bend J, Abele-Horn M, Baumann I, Bremer W, et al. CF lung disease-a German S3 guideline: module 2: diagnostics and treatment in chronic infection with Pseudomonas aeruginosa. Pneumologie (Stuttgart, Germany). 2018;72(5):347-92.
2. AmoClav. [Available from: https://www.gelbe-liste.de/produkte/beipackzettel_Amoclav-875-mg-125-mg-Filmtabletten.pdf/734afd5c-d352-4103-a592-2680a646f542.
3. AmoClavliquid. [Available from: https://www.gelbe-liste.de/produkte/beipackzettel_Amoclav-Trockensaft-125-31-25-mg-5-ml-Pulver-zur-Herstellung-einer-Suspension-zum-Einnehmen.pdf/720da084-17f1-4ace-8c31-ae5b951554e5.
4. AmoClavLiquid400. [Available from: https://www.gelbe-liste.de/produkte/beipackzettel_Amoclav-400-57-mg-5-ml-Trockensaft-Pulver-zur-Herstellung-einer-Suspension-zum-Einnehmen.pdf/467f2d27-bf0d-4b05-8d3f-52d1aa2068b7.
5. Infectomox. [Available from: https://data-storage.live/data/unsec/pb-infectomox-750saft.pdf.
6. Bodmann K-F, Grabein B, Kresken M. S2k guideline "Calculated parenteral initial treatment of bacterial infections in adults–update 2018", 2nd updated version: Foreword. GMS Infectious Diseases. 2020;8.
7. Cefpodoxime. [Available from: https://www.gelbe-liste.de/produkte/beipackzettel_Cefpodoxim-HEXAL-40-mg-5-ml-Pulver-zur-Herstellung-einer-Suspension-zum-Einnehmen.pdf/4497845f-4832-4ec5-aacd-d0a80e717cff.
8. Müller F-M, Bend J, Huttegger I, Möller A, Schwarz C, Abele-Horn M, et al. S3-Leitlinie „Lungenerkrankung bei Mukoviszidose ". Monatsschrift Kinderheilkunde. 2015;6(163):590-9.
9. AzythromycinLiquid. [Available from: https://www.fachinfo.de/static/lib/pdfjs/web/viewer.html?file=/fi/pdf/010322/azithromycin-ratiopharm-r-200-mg-5-ml#zoom=auto.
10. Erythromycin200. [Available from: https://data-storage.live/data/unsec/pb-infectomycin-200saft.pdf.
11. Erythromycin400. [Available from: https://data-storage.live/data/unsec/pb-infectomycin-400saft.pdf.
12. Erythromycin600. [Available from: https://data-storage.live/data/unsec/pb-infectomycin-600saft.pdf.
13. Clarithromycin125. [Available from: https://www.gelbe-liste.de/produkte/beipackzettel_Clarithromycin-1-A-Pharma-125-mg-5-ml-Granulat-zur-Herstellung-einer-Suspension-zum-Einnehmen.pdf/4964180c-d861-44b6-9a1a-1f5908d42803.
14. Clarithromycin250. [Available from: https://www.gelbe-liste.de/produkte/beipackzettel_Clarithromycin-1-A-Pharma-250-mg-5-ml-Granulat-zur-Herstellung-einer-Suspension-zum-Einnehmen.pdf/4ebf2a16-a9a6-4132-b36c-63f17b6f8dfb.
15. Co-trimoxazole. [Available from: https://www.gelbe-liste.de/produkte/beipackzettel_Cotrim-forte-ratiopharm-800-mg-160-mg-Tabletten.pdf/df28ec3a-8477-4d1e-92dd-5d9de40e0182.
16. Co-trimoxazoleLiquid200. [Available from: https://www.gelbe-liste.de/produkte/beipackzettel_Cotrim-K-ratiopharm-200-mg-5-ml-40-mg-5-ml-Suspension-zum-Einnehmen.pdf/a8af7b7e-939f-4128-b794-9aca49946070.
17. Co-trimoxazoleLiquid400. [Available from: https://www.gelbe-liste.de/produkte/beipackzettel_Cotrim-E-ratiopharm-400-mg-5-ml-80-mg-5-ml-Suspension-zum-Einnehmen.pdf/0ded57e2-8b70-40f8-954b-72ac38c8a911.
18. Doxycycline. [Available from: https://www.gelbe-liste.de/produkte/beipackzettel_Doxycyclin-100-1-A-Pharma-100-mg-Tabletten.pdf/0b91d543-6238-46d1-bc72-fae79e25b93d.
